# Cryo-EM structures of the mammalian endo-lysosomal TRPML1 channel elucidate the combined regulation mechanism

**DOI:** 10.1007/s13238-017-0476-5

**Published:** 2017-09-21

**Authors:** Sensen Zhang, Ningning Li, Wenwen Zeng, Ning Gao, Maojun Yang

**Affiliations:** 1 Ministry of Education Key Laboratory of Protein Science, Tsinghua-Peking Joint Center for Life Sciences, Beijing Advanced Innovation Center for Structural Biology, School of Life Sciences, Tsinghua University, Beijing, 100084 China; 2 State Key Laboratory of Membrane Biology, Peking-Tsinghua Center for Life Sciences, School of Life Science, Peking University, Beijing, 100871 China; 3 Institute for Immunology and School of Medicine, Tsinghua-Peking Joint Center for Life Sciences, Tsinghua University, Beijing, 100084 China

**Keywords:** mTRPML1, mucolipidosis type IV, structual comparisons, combined regulation mechanism

## Abstract

TRPML1 channel is a non-selective group-2 transient receptor potential (TRP) channel with Ca^2+^ permeability. Located mainly in late endosome and lysosome of all mammalian cell types, TRPML1 is indispensable in the processes of endocytosis, membrane trafficking, and lysosome biogenesis. Mutations of TRPML1 cause a severe lysosomal storage disorder called mucolipidosis type IV (MLIV). In the present study, we determined the cryo-electron microscopy (cryo-EM) structures of *Mus musculus* TRPML1 (mTRPML1) in lipid nanodiscs and Amphipols. Two distinct states of mTRPML1 in Amphipols are added to the closed state, on which could represent two different confirmations upon activation and regulation. The polycystin-mucolipin domain (PMD) may sense the luminal/extracellular stimuli and undergo a “move upward” motion during endocytosis, thus triggering the overall conformational change in TRPML1. Based on the structural comparisons, we propose TRPML1 is regulated by pH, Ca^2+^, and phosphoinositides in a combined manner so as to accommodate the dynamic endocytosis process.

**Electronic supplementary material:**

The online version of this article (doi:10.1007/s13238-017-0476-5) contains supplementary material, which is available to authorized users.

## Introduction

Mucolipidosis type IV (MLIV), first identified in 1974 (Berman et al., 1974; Bundey et al., 1974; Venkatachalam et al., 2015), is a severe autosomal recessive lysosomal storage disorder disease, with around one occurrence in every 40,000 people and 70 percent of the affected individuals are Ashkenazi Jewish (Bach, 2005; Bargal et al., 2001; Puertollano and Kiselyov, 2009). Classified as a mucolipidosis, MLIV exhibits a simultaneous lysosomal accumulation of granulated water-soluble materials and membranous lipid substances in the tissue and organelles of these patients (Bach, 2005; Zeevi et al., 2007). Children afflicted with MLIV often show psychomotor retardation, iron deficiency, retinal degeneration, and motor deficits in the early stage of their life and those who survive to adulthood usually have a shortened lifespan (Sun et al., 2000; Weitz and Kohn, 1988). Mutations in MCOLN1, a gene that encodes TRPML1 (mucolipin-1), have been found to cause MLIV (Bargal et al., 2000; Sun et al., 2000). TRPML1 is characterized as a non-selective, cation-permeable channel (Venkatachalam et al., 2015), which might mediate the release of Ca^2+^ from lysosomal or late endosomal lumen into the cytosol (Cheng et al., 2010; Cheng et al., 2015), thus regulating membrane trafficking, lysosomal biogenesis, and signal transduction (Colletti and Kiselyov, 2011; Samie et al., 2013; Wang et al., 2014).

TRPML1 belongs to the family of transient receptor potential mucolipin channels (TRPML channels) (Benemei et al., 2015; Clapham, 2003; Clapham et al., 2001; Flockerzi, 2007; Gees et al., 2012) and this family is the only cation channels known so far to be localized and function in the digestive tract (Colletti and Kiselyov, 2011). TRP channels consist of six sub-families, TRPA, TRPC, TRPM, TRPP, TRPV, and TRPML, classified by sequence homology (Han and Wang, 2008; Minke, 2006; Pedersen et al., 2005; Ramsey et al., 2006). Meanwhile, according to topology similarities, they are divided into two distinct groups (group 1 and group 2) (Venkatachalam and Montell, 2007). Group 2 contains two members, TRPP and TRPML (Qian and Noben-Trauth, 2005), as they have a large extracellular loop between transmembrane segments 1 and 2, which differs significantly from group 1 TRP subfamily (Grieben et al., 2017; Shen et al., 2016; Wilkes et al., 2017). Structural studies empowered by the great improvement of single particle cryo-EM method have revealed significant differences between the two groups. Recently, three individual research groups have reported the structures of TRPP2 determined by cryo-EM with three different states named TRPP2CL (closed) (Grieben et al., 2017; Shen et al., 2016), TRPP2MI (multi-ion), and TRPP2SI (single-ion) (Wilkes et al., 2017) and these distinct structures were presented to elucidate the possible gating and regulatory mechanisms of group 2 channels.

Unlike other plasma ion channels, TRPML1 predominately resides on late endosome and lysosome membrane during endocytosis, and also temporarily resides on cell surface plasma during exocytosis (Cheng et al., 2010). The di-leucine motifs in both N- and C-terminus mediate its localization to late endosome and lysosome (Venkatachalam et al., 2015; Vergarajauregui and Puertollano, 2006). Two models of regulatory mechanism were proposed in terms of how to ensure proper level of the channel activity. One model is the subcellular-compartment-dependent regulation by phosphoinositide. In this model, different phosphoinositide isoform may regulate the channel activity through direct binding to the N-terminus lipid-interacting domain (spanning amino acid residues 42–62). PI(3,5)P2, a specific late endosome/lysosome phosphoinositide, may activate TRPML1 channel during membrane trafficking and lysosome biogenesis (Dong et al., 2010a; Waller-Evans and Lloyd-Evans, 2015). On the contrary, a plasma membrane specific phosphoinositide, PI(4,5)P2 may function to inhibit TRPML1, thus shutting down excess Ca^2+^ influx from extracellular matrix to cytosol, which in turn could lead to cell death (Zhang et al., 2012). The other model of regulatory mechanism is the pH and Ca^2+^ dual regulation of the PMD. This dual regulation is achieved through different pH and Ca^2+^ levels in different organelles. Late endosome have 0.5 mmol/L Ca^2+^, which is about 1000 fold to the concentration of Ca^2+^ in cytosol, and an acidic pH of 5.5–6.0. Meanwhile, lysosomes have a more acidic pH of 4.5–5.0 to supply acid environment for native hydrolases (Appelqvist et al., 2013) and 0.5–0.6 mmol/L Ca^2+^. The extracellular side of the plasma membrane has a neutral pH of 7.2 to 7.4 and an obviously higher concentration of Ca^2+^ (1.8–2.0 mmol/L) (Dong et al., 2010b). High concentration of Ca^2+^ was shown to inhibit the current of TRPML1 (V432P) channel in whole-cell patch-clamp recording (Li et al., 2017; Xu et al., 2007). In late endosome and lysosome, luminal Ca^2+^ inhibition of TRPML1 attenuates following the pH decrease from 7.4 to 5.5 and 4.5, respectively. Minghui Li et al. solved the structures of this PMD at different pH conditions, which provides structural basis of TRPML1’s regulation by proton and Ca^2+^ and suggested that the protonation of 12 aspartates in the luminal pore at different pH conditions is responsible for the attenuation (Li et al., 2017).

However, elucidating how TRPML1 responds to different pH, Ca^2+^, and phosphatidylinositol during endocytosis and exocytosis has been hampered by a lack of overall structure information (Waller-Evans and Lloyd-Evans, 2015; Zhang et al., 2012). Here, using single-particle electron cryo-microscopy (cryo-EM) method, we determined the structure of mTRPML1 in lipid nanodiscs at a resolution of 5.4 Å. Moreover, in order to mock the subcellular locations of TRPML1 and interpret compartment regulation mechanism, we solved the structures of mTRPML1 in Amphipols at resolutions of 5.8 (state 1), 7.4 (state 2), and 7.7 Å (state 3), respectively. Structure comparisons between these three conformations of TRPML1 in Amphipols have revealed the potential channel gating mechanism and sheds light on the regulation of compartment specificity of TRPML1 channel.

## Results

### Structure determination of mTRPML1

**Figure 1:**
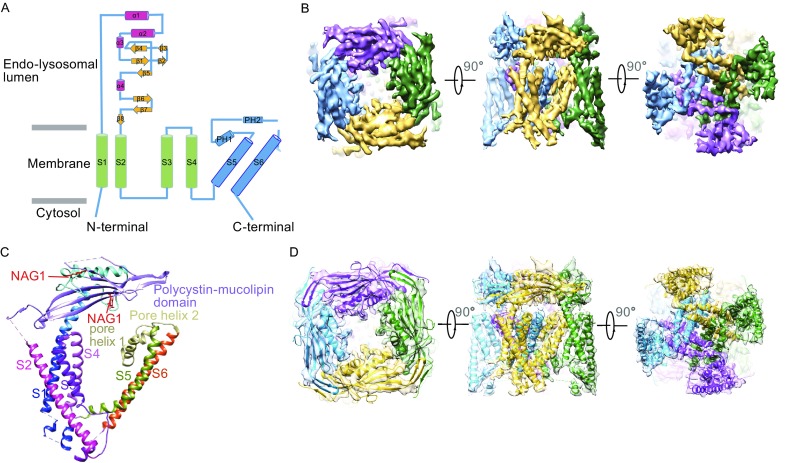
**Overall structure of mTRPML1**. (A) Secondary-structural organization of mTRPML1 showing VSD (cyan), PMD (pink and yellow), and pore region domain (blue). Cylinders, arrows indicate helices, β-strands, respectively. N-terminus and C-terminus face cytosol and PMD locates in the endo-lysosomal lumen. (B) Cryo-EM density map of mTRPML1 at a resolution of 5.4 Å with each subunit color-coded. Three views are shown from the endo-lysosomal lumen, side and cytosol. (C) Ribbon diagram representation of mTRPML1 subunit with different domains colored as shown. (D) mTRPML1 model superimposed with the cryo-EM map displayed from different views as panel (B) showed. See also Figs. S1 and S2

The full length mTRPML1 was expressed and purified from HEK293F cells through Strep-tag II affinity chromatography in the buffer containing 5 mmol/L CaCl_2_ at pH 7.4. In order to visualize TRPML1 in a native lipid-like environment, we reconstituted mTRPML1 into lipid nanodiscs, which consist of the channel protein (TRPML1), membrane scaffolding protein (MSP2N2), and soy extract polar (Figs. S1, S2, and METHODS) (Gao et al., 2016; Shen et al., 2016). Using single particle cryo-EM method, we determined the structure of mTRPML1 at a resolution of 5.4 Å (Figs. 1B, 1D, S2, and STAR METHODS). As mTRPML1 resembles human TRPML1 in a primary sequence similarity up to 91% (Fig. S3), we performed a direct and rigid body fitting of the human crystallographic PMD structure into the cryo-EM density map (Li et al., 2017). Based on the second structure prediction from the I-TASSER server (Zhang, 2008), we build the poly-alanine model encompassing the PMD, transmembrane domain, a small part of the N-terminus and C-terminus. Despite the usage of full-length protein of mTRPML1, densities accounting for the N-terminus (1–35aa) and C-terminus (544–580aa) regions are not apparent in the cryo-EM map and the build model excludes these regions.

As TRPML1 mainly functions in the dynamically endocytosis/exocytosis process, the structure and function of the channel might be different in order to accommodate to various environment. With the aim of seizing possible different states of TRPML1, we reconstituted mTRPML1 in Amphipols after purification with n-dodecyl-β-D-maltoside (DDM). Interestingly, after rounds of 3D classification with sufficient tests, three conformational states of mTRPML1 were identified at resolutions of 5.8 (state 1), 7.4 (state 2), and 7.7 Å (state 3) (Fig. S4), respectively. These different states led us to speculate that those might result from different subcellular locations of mTRPML1 during endocytosis/exocytosis pathway and could shed light on the regulation mechanism of TRPML1.

### Overall structure of mTRPML1

Structural comparison between mTRPML1 in lipid nanodiscs and in Amphipols (state 1) (Figs. 1B, 1D, S4, and S5) reveals that these two structures exhibit indistinguishable channel features due to lower resolution, except that the mTRPML1 in Amphipols lacks the densities for N-terminus, C-terminus and S2–S3 loop. The emerging N-terminus (36–58) contains the phosphatidylinositol binding sites (Zhang et al., 2012). It appears that soy extract polar lipid helps to stabilize the channel integrity and helps us obtain the higher resolution structure. Four mTRPML1 subunits form a tetrameric architecture with each subunit consisting of six transmembrane helices and a re-entrant pore loop intervening transmembrane helix S5 and S6 (Fig. 1A and 1C). The voltage-sensing domain (VSD) consists of the S1 to S4 helices and is similar to other voltage-gated ion channels (VGICs) (Long et al., 2005) and TRP channels (Cao et al., 2013; Huynh et al., 2016; Paulsen et al., 2015) (Fig. S6).

Apart from this canonical and conserved transmembrane region, mTRPML1 contains a large luminal/extracellular PMD between the transmembrane helix S1 and S2 (Fig. 1A and 1C). The PMD resides upon the lipid bilayer of the plasma membrane, endosome membrane and lysosome membrane. This tightly packed tetrameric ring structure consists of two long α-helices (α1 and α2), two short α-helices (α3 and α4), eight β-strands and three loops (Fig. 1A and 1C) (Li et al., 2017). Accounting for 1/3 of the ion channel mass, this PMD only exists in TRPML and TRPP families, and it contributes not only to the channel assembly by homotypic interaction but also to the allosterically regulation and channel gating. Furthermore, the linker between S2 and S3 protrudes toward intracellular, which is different from TRPP2 (Grieben et al., 2017; Shen et al., 2016; Wilkes et al., 2017). Interestingly, this long extension lays close to the N-terminus phosphatidylinositol binding sites (36–58) and C-terminus helix (536–543). High resolution structure will elaborate the possible function of this intracellular interaction.

Glycosylation of TRPML1 is an indispensable process in channel function (Hofherr et al., 2014; Kiselyov et al., 2005; Zeevi et al., 2007). mTRPML1 has four proposed N-glycosylation sites (Asn159, Asn179, Asn220, Asn230) and the modification occurs at the consensus NX(T/S) motif (X stands for any amino acid) (Wang et al., 2014). The density shows that two asparagine residues (Asn159, Asn230) are obviously N-glycosylated (Fig. S7). Asn179 and Asn220 are adjacent to the disordered region and are not resolved in this structure. It remains intriguing whether N-glycosylation will trigger the conformational change or whether different states will adopt different patterns of N-glycosylation.

### Continuous connection between S1 and PMD

**Figure 2:**
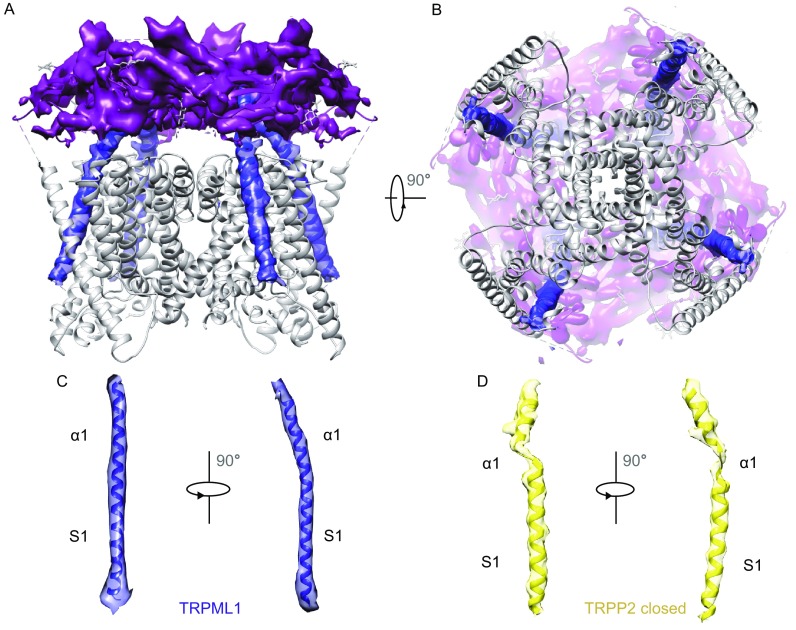
**Continuous connection between S1 and α1**. (A) Schematic representation of TRPML1 with the PMD region shown in cryo-EM density map (pink) and the transmembrane region shown as ribbon model. Continuous connection between S1 and α1 was shown in cryo-EM density map (blue). Side view was shown in Fig. 2A. (B) Bottom view of TRPML1. (C) S1 and α1 helices of mTRPML1 were fitted into the cryo-EM map. (D) S1 and α1 helices of TRPP2-CL were fitted into the cryo-EM map. The different connection of S1 and α1 between mTRPML1 and TRPP2-CL reveals different regulation mechanism. See also Figs. S5 and S7

The interaction between different subunits of TRPP2 helps maintaining the channel stability and this cooperativity between neighboring chains are contributed by the interaction between their PMD and the pore turret of adjacent chain. Meanwhile, the interaction between different PMDs and the interaction between S3-loop-S4 regions with the adjacent PMD also contribute to the tetramer formation (Grieben et al., 2017; Shen et al., 2016). In our map, however, there is no such diverse interaction between the PMD and the transmembrane domain. It is obvious when compared with mTRPML1 that TRPP2 harbors a more compact structure (Fig. 2A). Interestingly, in TRPML1, the α1 helix of PMD and S1 of transmembrane domain form a continuous α-helix (Fig. 2A–C), whereas in TRPP2 the corner bending linker between S1 and α1 is a short loop (Fig. 2D). This unique helix extension between S1 and α1 is the only rigid joint between PMD and transmembrane domain. This integrated helix might sense the movement from the luminal pore during channel open/close process and propagate this conformational change to the transmembrane segments and cytoplasmic through the continuous α helix.

### Ion translocation pathway in mTRPML1

**Figure. 3:**
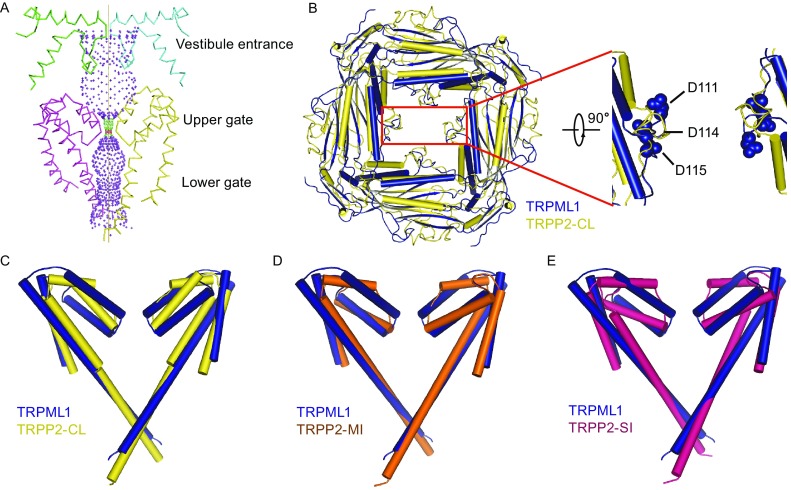
**Ion permeation pathway of mTRPML1 and comparison with TRPP2**. (A) Ion permeation pathway of mTRPML1 was shown along with two diagonally opposed promoters using HOLE program (Smart et al., 1996). Ion permeation pathway contains the vestibule entrance, the upper gate, and the lower gate from top to bottom as indicated (A). (B) Comparison of the PMD between mTRPML1 and TRPP2 aligned by β strands and viewed from the endo-lysosome lumen (B). A close-up view of the luminal pore loop was shown with two diagonally opposed promoters and residues (D111, D114, and D115) was shown as blue balls. (C) Superposition of the filter region and lower gate between mTRPML1 (yellow) and TRPP2 closed (blue). (D) Superposition of the filter region and lower gate between mTRPML1 (yellow) and TRPP2-MI (orange). (E) Superposition of the filter region and lower gate between mTRPML1 (yellow) and TRPP2-SI (pink). These comparisons (C–E) indicate the difference between TRPML1 and TRPP2. See also Figs. S2, S4, and S5

Similar to TRPP2, the ion translocation pathway of TRPML1 consists of three constrictions/gates: a vestibule entrance in the luminal/extracellular PMD, a selectivity filter in the pore region intervening S5 and S6, and a lower gate comprised of distal end of S6 (Fig. 3A) (Grieben et al., 2017; Shen et al., 2016). Compared with the ion permeation pathway from group 1 TRP family, group 2 TRPML1 channel adopts the luminal/extracellular loop as a potential physical substrate for luminal/extracellular stimuli to gate the channel (Wilkes et al., 2017). The electronegative luminal pore loop in TRPML1 extends downward toward the luminal/extracellular entry, whereas in TRPP2 it faces upward toward the ion-selectivity filter (Fig. 3B). Previous study suggested that the protonation of 12 aspartate amino acids in the luminal loop accounts for the attenuation of Ca^2+^ block at different pH condition (Fig. 3B), thus forming a vestibule entrance gate to mediate ion translocation (Li et al., 2017). The selectivity filter spanning residues 446–490 is a relatively conserved region between TRPML1 and TRPP2. The residues Asn471 and Asn472 in this region could coordinate with permeating cations. Compared with different states of TRPP2 (Fig. 3C–E), the lower constrictions of mTRPML1 are a little wider than the homologous sites (Asn643 and Leu677) of TRPP2-CL and narrower than TRPP2-MI and TRPP2-SI when measured Cα-Cα. Moreover, TRPML1 exhibits great conformational change in the filter region when compared with TRPP2-SI (Fig. 3E). These differences indicate the diversity of TRP channels.

### Three different states of mTRPML1 in Amphipols

**Figure 4:**
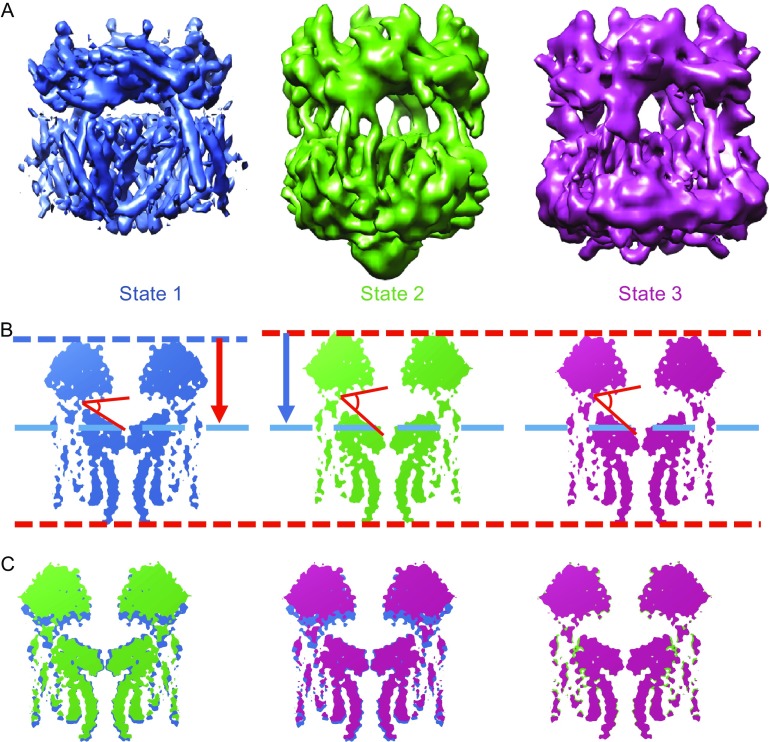
**Three different states of mTRPML1 in Amphipols A8–35**. (A) Three different mTRPML1 models were superimposed onto the cryo-EM map with side views. State 1, 2, and 3 were colored with blue, cyan, and magenta as showed. Three states were filtered to 8 Å (at 3σ contour level). (B) A vertical section perpendicular to the transmembrane region in three states was carried out. Intuitively, the distance between PMD and filter region in state 2 and 3 were larger than state 1, as indicated by red (state 1) and blue arrows (state 2 and 3). Moreover, an amphipathic tunnel between PMD and transmembrane domain expands compared with state 1. (C) Comparison of the vertical section between different two states. As indicated, State 2 and 3 harbors a longer structure along the vertical section. The difference between state 2 and 3 was slightly, which lies mainly in transmembrane region. See also Figs. S1, S4, and S5

3D classification of TRPML1 particles in Amphipols reveals three distinct conformational states of mTRPML1 with the resolution of 5.8 (state 1), 7.4 (state 2), and 7.7 Å (state 3), respectively (Figs. 4A and S4). Based on the architecture of mTRPML1 in nanodiscs, we reconstruct these three structures by rigid body fitting. In all three states, densities of glycosylation of Asn159 and Asn230 are visible while putative Asn220 and Asn179 glycosylation sites are not clear due to the disordered regions.

To gain further insight into the differences between these three states, we transform the constructed model structures into density maps and slice a vertical section across the transmembrane regions (Fig. 4B). Intuitively, compared with state 1, state 2 and 3 harbor a longer structure in the vertical direction. An amphipathic tunnel between pore filter region and PMD enlarges the cave pocket and contributes to the vertical extension (Fig. 4C). This tunnel enlargement was due to the movement of the outer PMD and accompanied by a twist between VSD region and pore filter region. The main difference between state 2 and state 3 lies in the transmembrane filter region with a slight twist occurring in the helix S5 and S6 (Fig. 4C).

**Figure 5:**
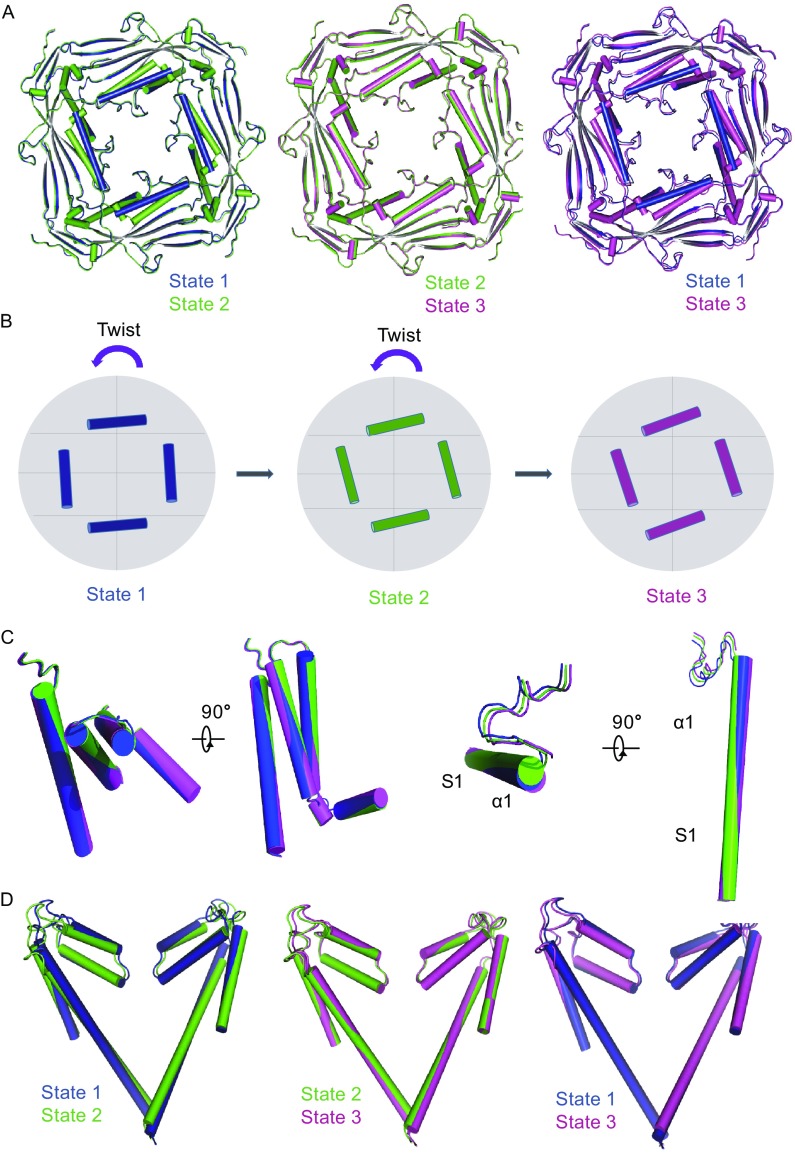
**Comparison between three states**. (A) Comparison of the PMD between three states aligned by the β sheets. Superposition of the PMD reveals an anticlockwise twist in the helices regions perpendicular to the transmembrane region, especially when sate 2 or 3 compared with state 1. (B) A schematic diagram depicts the anticlockwise twist from state 1 to state 3. (C) Comparison of the VSD and S1-α1 regions with two views as indicated. A twist in these regions helps to propagate the conformational change during endocytosis/exocytosis. (D) Superposition of the filter regions in three states reveals a twist and displacement. Both the upper and lower gate of state 2 and state 3 is larger than state 1. Moreover, State 3 has a wider lower filter gate, while State 2 has a wider upper pore restriction. See also Figs. S1, S2, S4, and S5

Single particle cryo-EM offers the opportunity to explore the dynamic conformational changes under different conditions. The PMD of mTRPML1 serves as a “plate” like region to sense physical or chemical stimuli from the endo-lysosomal lumen and its electronegatively charged luminal pore plays an indispensable role in the attenuation of Ca^2+^ block. State 3 has a similar luminal pore diameter with state 2 and is a little wider than that in state 1 when measured Cα-Cα in the same residue of the pore loop (Fig. 5D). Superposition of the PMD in these three states revealed that this region undergoes an anticlockwise twist relative to the transmembrane region from state 1 to state 3 when viewed from the luminal/extracellular side (Fig. 5A). Moreover, this twist is visible in the second α helix when compared with all the three states (Fig. 5A and 5B). Interestingly, we perceive that state 2 and 3 exhibit a “move-upward” motion in the direction perpendicular to the membrane when compared with state 1 (Fig. 4C). This move-upward motion in the luminal/extracellular loop appears to trigger a swivel of transmembrane regions including the selective filter pore and provide us a clue to investigate the channel gating mechanism.

Similar to other TRP channel structures, the central ion permeation pathway of the TRPML1 exhibits two major canonical constrictions gates. We have discussed the difference in lower gate between state 1 mTRPML1 channel and other TRPP2 channels above. Measurement of the upper pore helix gate in three mTRPML1 states shows that state 1 seems to adopt a narrow upper gate and state 2 has the widest upper gate. Continuing down the pore, when measured the distances between Cα-Cα in the same residue of the lower gate, state 1 still exhibit a narrow gate whereas state 3 has the widest lower gate. Taken together, state 1 seems to be the narrowest ion permeation state and represents the close state. State 3 has a wider lower filter gate, while state 2 has a wider upper pore restriction (Fig. 5D). This comparison is consistent with the three states of TRPP2, yet the more opened state TRPP2-SI has a wider lower gate than TRPP2-MI but exhibits a narrow pore helix upper gate to TRPP2-MI (Grieben et al., 2017; Shen et al., 2016; Wilkes et al., 2017). Superposition of state 1 and 3 in the transmembrane helix region resulted in a conformational twist and shift in both VSD and filter region, and this swivel is accompanied with conformational change in the outer PMD (Fig. 5C). In addition, comparison of the S1 and α1 helix region between all three states is consistent with our hypothesis that the continuous helix twist could mediate conformational change transduction through the outer PMD to the transmembrane region (Fig. 5C).

### Structure interpretation of mucolipidosis type IV pathogenesis

MLIV is a severe neurodegenerative lysosome storage disorder disease caused by mutations in *MCOLN1* gene, which locates on chromosome 19p13.2-p13.3 (Zeevi et al., 2007). There are more than 20 *MCOLN1* mutations identified in MLIV patients. Among them, two major founded *MCOLN1* mutations in Ashkenazi Jewish patients account for 95% of disease-associated alleles (Bargal et al., 2001; Venkatachalam et al., 2015). The most common one is a splice site mutation g.5534A→G that comprises 72% patients in Ashkenazim Jewish population resulting in aberrant splicing and truncated unstable mRNA species (Wakabayashi et al., 2011). Another common mutation comprising 23% patients is caused by a ∼6 kb deletion spanning the first six exons as well as a 12 bp deletion of exon 7 (Zeevi et al., 2007). Other mutations include nonsense mutations, missense mutations and in-frame deletion (Everett, 2011).

**Figure 6:**
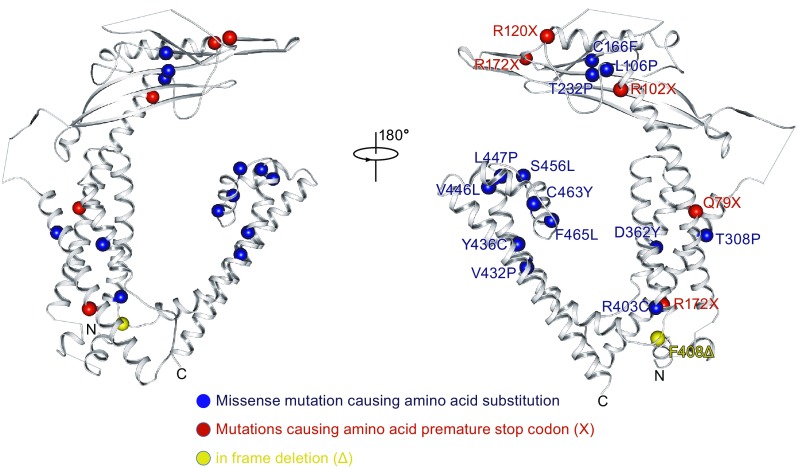
**Structural interpretation of mucolipidosis type IV pathogenesis**. Mapping of human mucolipidosis type IV mutations onto the structure of an mTRPML1 subunit. Note that PMD and pore filter region are mutation hot-spots. Blue ball indicates missense mutation, red balls indicate mutations causing amino acid premature stop codon (X) and yellow ball indicates in frame deletion (Δ). See also Figs. S2, S4, and S5

Structural elucidation of mTRPML1 lays the foundation to understand the potential mechanism of pathogenic mutations. All of the amino acid change mutations can be mapped onto the resolved structure of mTRPML1 (Fig. 6). Interestingly, no pathogenic mutations have been identified in the N- and C-terminus to date, suggesting these regions has less relevance to human disease. Among the mapped disease mutations, PMD and pore helix region harbor 6 and 5 residues, respectively, demonstrating their significant role in channel function (Fig. 6). Mutations R102X and L106P localizes in the α1 helix of PMD, which could perturb the signal propagation from the transmembrane region when mTRPML1 undergoes conformational changes. The mutations (C166F, R172X, T232P) in β sheet region of PMD might destabilize the tightly packed tetragon to affect the channel function. Changes in S5 helix (V432P, Y436C, V446L, and L447P) and re-entrant pore loop (S456L, C463Y, and F465L) may affect the selectivity filter in the ion permeation pathway and potentially disrupt opening of the channel. Moreover, mutations from in-frame deletion of TRPML1 greatly influence the channel integrality. F408Δ, an amino acid deletion in the linker helix between S4 end and S5, was postulated as a semi-functional form of TRPML1 (Qian and Noben-Trauth, 2005) (Fig. 6). F408Δ is intriguing because it preserves function in normal neurological development despite high ophthalmologic and gastrin defects (Chen et al., 2014). Splice mutation c.1406 A→G result in amino acid deletion from 454 to 469, which spans the pore loop region, and this in-frame deletion disrupts pore architecture of the channel.

## Discussion

Although biological studies of TRPML1 have been extensively carried out in the past (Ahuja et al., 2016; Cheng et al., 2010; Colletti and Kiselyov, 2011; Waller-Evans and Lloyd-Evans, 2015; Wang et al., 2014), the molecular mechanism of how the TRPML1 channel gating remains elusive, largely due to the lack of detailed structural information. In the present study, we embarked on solving the structure of mTRPML1 and obtained the different conformational states in nanodiscs and Amphipols. *In vitro* reconstruction of mTRPML1 in lipid nanodiscs could represent the native and comprehensive cellular environment to a large extent. Soy polar extract is a natural product generated from soybean and this hockey-puck-like structure mimic the native lipid environment (Gao et al., 2016; Ritchie et al., 2009). Phosphatidylinositol, especially the PI(4,5)P2 from the soybean polar could inhibit the channel activity and helps stabilize the structure through the N-terminus binding site (Zhang et al., 2012). In addition, we added 5 mmol/L CaCl_2_ during protein purification and nanodiscs reconstruction. Therefore, we speculated this lipid chimeric structure represents a channel-closed state. Whereas in the reconstruction of mTRPML1 in Amphipols A8–35, we did not add extra CaCl_2_ and the purification buffer was maintained at pH 7.4, which could not provide a sustaining Ca^2+^ inhibition on PMD. High-level overexpression of TRPML1 will also drive TRPML1 from endo-lysosome to plasma membrane (Zhang et al., 2012). Hence, in the initial detergent (DDM) extraction step, mTRPML1 in different organelles with different conformations were obtained. It is possible that the three different structural states represent different “energy” states, or intermediate states, which can be converted to different functional states by additional regulatory factors, such as extracellular pH, phosphatidylinositol and Ca^2+^. We speculate therefore that State 1 may represent the channel-closed state in the environment of pH 7.4 when TRPML1 resides on the plasma membrane and State 2, 3 adopt relatively opened conformation, which exhibited as transition states involved in the endocytosis trafficking process.

**Figure 7:**
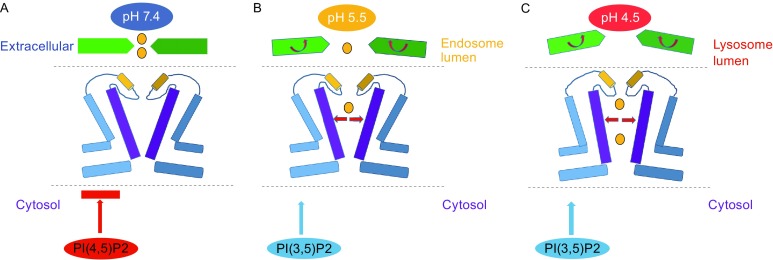
**Models of TRPML1 gating mechanism**. (A) Proposed gating mechanism of TRPML1 in plasm membrane. Due to the high concentration of Ca^2+^ and the inhibition of plasma membrane PI(4,5)P2, TRPML1 exhibits in a closed state. (B) Proposed gating mechanism of TRPML1 in endosome. Endo-lysosomal specific PI(3,5)P2 binds directly to the N-terminus of TRPML1 and helps activate channel. Moreover, in the endosomal lumen, the pH was about 5.5 and it will attenuates the Ca^2+^ inhibition. Taken together, TRPML1 undergoes a conformational change and exhibits a relatively open state. (C) Proposed gating mechanism of TRPML1 in lysosome. Compared with the environment in endosome, the pH in decrease to about 4.5 and it will attenuates the Ca^2+^ inhibition to a large extend. Plus the activation of PI(3,5)P2, TRPML1 exhibits as an open state in lysosome. See also Fig. S2–S7

Based on the fact that TRPML1 can be regulated by different pH, Ca^2+^ concentration and phosphoinositides, we propose a combined regulation mechanism according to the structures of mTRPML1 in lipid nanodiscs and Amphipols. When TRPML1 is located on the plasma membrane, this channel will act as a close state (Fig. 7A). High concentration of Ca^2+^ (1.8–2.0 mmol/L) on the extracellular side of the plasma membrane will inhibit channel activity through binding to the luminal pore asparagines (D111, D114, and D115). Moreover, plasma membrane specific phosphoinositide PI(4,5)P2 will interact with N-terminus positively charged residues (R42, R43, and R44) (Zhang et al., 2012) and keep the channel at a close state. When TRPML1 participates in the endocytosis process in late endosome and lysosome, this channel will represent open states (Fig. 7B and 7C). In late endosome, pH decreases from 7.2 to 5.5, which is close to the p*K*_a_ of the aspartate side chain, thus decreasing the aspartate negative charges and attenuating the Ca^2+^ block. Meanwhile, PI(3,5)P2, an endo-lysosome specific phosphoinositide will bind to the N-terminus residues (R61 and K62) of TRPML1 and activate the channel (Zhang et al., 2012) (Fig. 7B). As depicted in our study, the whole channel undergoes a conformational change to accommodate the specific environment. The tetragonal PMD adopt a “move-upward” motion upon the transmembrane region and the amphipathic cave pocket between pore filter region and PMD could enlarge (Fig. 4B). Furthermore, this vertical stretch is accompanied by twists of the VSD and pore filter regions through continuous joint between S1 and α1 helix (Fig. 5C). The distance between upper and lower gate in the pore filter regions becomes larger, thus inducing channel in a relatively open state (Figs. 7B and 5D). When TRPML1 resides in the lysosome, this channel will turn into open state. As the pH of lysosome is about 4.5, more aspartates could be protonated and attenuate the Ca^2+^ block to a large extend. The PMD may also undergo a “move-upward” motion and the vertical stretch is larger compared with motion in late endosome (Fig. 7C). Moreover, PI(3,5)P2 will also active the channel and the transmembrane regions undergo twist in both VSD and pore filter regions (Fig. 7C). The lower gate will become larger than that in late endosome, while the upper gate is narrow compared with that in late endosome (Figs. 7B, 7C and 5D). Interestingly, in both late endosome and lysosome, the PMD undergoes an anticlockwise twist compared with the closed form. This is similar to TRPV and NOMPC channels as the ARD region assembly rotates relative to the transmembrane region during channel gating (Huynh et al., 2016; Jin et al., 2017).

The heterogeneity of TRPML1 combines the cellular organelle compartment with different regulatory factors, including pH, Ca^2+^ and phosphoinositide. Our structural analyses indicate that the TRMPL1 protein is regulated in a combined manner with these regulatory factors. High-resolution structure of TRPML1 will further replenish the regulation mechanism during endocytosis and provide a chance to better understand their roles in mucolipidosis pathogenesis.

## Materials and Methods

### Cell culture

HEK293F cells were cultured in SMM 293-TI medium (Sino Biological Inc.) supplemented with 1× penicillin/streptomycin (Solarbio) at 37°C with 8% CO_2_.

### Transient transfection

For protein expression, mTRPML1 was cloned in frame with a C-terminus strep-tag II (WSHPQFEK) into the plasmid pcDNA3.1 (-). Transient transfection was performed to heterogeneously express the target protein. In brief, for one liter culture of HEK293F cells, 1 mg plasmids was pre-incubated with 4 mg 25-kDa linear polyethylenimine (PEI) (Polysciences) in 50 mL fresh medium for 30 min prior to adding the mixture to cells. The transfected cells were cultured for 48 h before harvesting.

### Protein purification and reconstructed in Amphipols A8–35

For each batch of protein purification, two liters of transfected HEK293F cells were harvested by centrifugation at 3,000 ×*g*. Cell pellets were resuspended in lysis buffer containing 20 mmol/L Hepes, pH 7.4, and 150 mmol/L NaCl, 1 μg/mL leupeptin, 1.5 μg/mL pepstatin, 0.84 μg/mL aprotinin, 0.3 mmol/L PMSF and lysed by sonication for 5 min. Cell membrane was pelleted after a 100,000 ×*g* ultracentrifugation for 1 h. Membrane was resuspended in buffer containing 20 mmol/L Hepes, pH 7.4, 150 mmol/L NaCl, 2 mmol/L DTT, and 1% (*w*/*v*) DDM (Anatrace) for 2 h with gentle rotation at 4°C. After ultra-centrifugation at 100,000 ×*g* for 20 min, the supernatant was incubated with Strep-Tactin Sepharose (IBA) for 1 h with gentle rotation at 4°C. The resin was washed extensively with wash buffer containing 20 mmol/L Hepes, pH 7.4, 150 mmol/L NaCl, 2 mmol/L DTT, 0.02% (*w*/*v*) DDM. The target mTRPML1 protein was eluted with wash buffer plus 5 mmol/L D-Desthiobiotin (IBA). The eluted protein was then mixed with Amphipols A8–35 at 1:3 (*w*/*w*) with gentle agitation for another 4 h. Detergent was removed with Bio-Beads SM-2 at 4°C overnight (15 mg per 1 mL channel/detergent/amphipols mixture). The reconstitution mixture was cleared by centrifugation before applied to size-exclusion chromatography (Superpose-6 10/300 GL, GE Healthcare) in buffer containing 20 mmol/L Hepes, pH 7.4, 150 mmol/L NaCl, 2 mmol/L DTT). The peak corresponding to tetrameric mTRPML1 channel reconstituted in Amphipols A8–35 was collected for further cryo-microscopy analysis.

### Protein purification and reconstructed in lipid nanodiscs

A similar protocol was used to purify mTRPML1 in nanodiscs during membrane extraction and affinity chromatography, except that 5 mmol/L CaCl_2_ was added in all buffers throughout purification since calcium was reported to stabilize TRPML1 channels (Li et al., 2017). Membrane scaffold protein MSP2N2 was expressed and purified from *E*. *coli* (Civjan et al., 2003; Ritchie et al., 2009) and lipid nanodiscs was reconstructed as described (Gao et al., 2016). In brief, soybean polar lipid extract (Avanti) dissolved in chloroform was dried under an argon stream and residual chloroform was further removed by vacuum desiccation (3 h). 10 mmol/L lipids stock was prepared by resuspending dried lipids in a buffer containing 20 mmol/L HEPES (pH 7.4), 150 mmol/L NaCl, 14 mmol/L DDM via bath sonication. Purified mTRPML1 protein (0.7 mg/mL) solubilized in 0.5 mmol/L DDM was mixed with MSP2N2 (3 mg/mL) and the soybean lipid stock at a molar ratio of 1:2:200, followed by incubate on ice for 30 min. Bio-beads SM2 (30mg per 1mL reconstitution mixture; Bio-Rad) were added to remove detergents and initiate lipid nanodiscs reconstitution at 4°C for 1 h with gentle rotation. Bio-beads were then removed and the reconstitution mixture was cleared by centrifugation before subsequent separation on a Superose 6 column (GE Heath Care) in buffer (20 mmol/L HEPES, 150 mmol/L NaCl, pH 7.4, 5 mmol/L CaCl_2_). The peak corresponding to mTRPML1 channel reconstituted in lipid nanodiscs was collected for electron cryo-microscopy analyses.

### Cryo-electron microscopy

The cryo-EM grids were prepared using Vitrobot Mark IV (FEI) at 4°C and 100% humidity. For samples of both mTRPML1 amphipols and nanodiscs, 4 μL aliquots of samples at concentrations of 0.5–0.7 mg/mL were applied onto glow-discharged holey carbon grids (Quantifoil R1.2/1.3). After a waiting time of 3 s, the grids were blotted for 1–2 s and plunged into liquid ethane for quick freezing.

The grids were screened on a Tecnai Arctica microscope (FEI) operated at 200 kV using a Falcon II direct electron detector (FEI). The qualified grids were transferred into a Titan Krios microscope (FEI) operated at 300 kV for data acquisition. Images were recorded using a K2 submit direct electron detector (Gatan) in a counting mode at a nominal magnification of 22,500×, corresponding to a calibrated pixel size of 1.32 Å at object scale, and with defocus ranging from 1.7–2.6 μm. Date acquisition were performed semi-automatically using UCSF-Image4 (Li et al., 2015) in a movie mode, with a dose rate of 8.2 counts (10.9 electrons) per pixel size per second for a total exposure time of 8 s. Each micrographs were stored as a movie stack with 32 frames.

### Image processing

2,565 and 3,944 micrographs (movie stacks) are collected for mTRPML1 amphipols and mTRPML1 nanodiscs, respectively (Figs. S2 and S4). For both batches of micrographs, global motion correction was first applied to all 32 frames using MOTIONCORR1 (Li et al., 2013), resulting global motion-corrected frame stacks and summed micrographs. The global motion-corrected stacks (the first 2 frames were discarded) were further processed by sub-region motion correction and dose weighting using MOTIONCORR2 (Zheng et al., 2017), generating summed micrographs with or without dose weighting. CTFFIND4 (Rohou and Grigorieff, 2015) was used to estimate the contrast transfer function (CTF) parameters and produce the CTF power spectrum on basis of summed micrographs from MOTIONCORR2. 2,565 and 3,678 micrographs were selected for further processing by screening summed micrographs and power spectra using SPIDER (Shaikh et al., 2008). Particles were auto-picked on summed micrographs from MOTIONCORR1 using RELION2.0 (Kimanius et al., 2016). For dataset of mTRPML1 amphipols, ~4,000 particles were manually picked in advance, and processed by 2D classification using RELION1.4 (Scheres, 2012). The resulting 2D averages were served as the templates for particle picking. For dataset of mTRPML1 nanodiscs, selected 2D averages from final 2D classification of mTRPML1 amphipols were used as the templates for particle auto-picking. For both batches of samples, two rounds of 2D classifications were performed to exclude noise and other bad particles. 783 K and 874 K particles from qualified 2D averages were selected for further 3D analysis. Ahead of 3D classification, a round of refinement was applied on the whole particles sets using RELION1.4. The particles were re-centered and processed by several rounds of 3D classification with tighter particle mask applied and C4 symmetry imposed using RELION1.4 (Figs. S2 and S4). To separate particles with different conformations and further exclude noise and bad particles more thoroughly, a cascade of 3D unsupervised or supervised classification with different combinations of parameters were tested.

For sample of mTRPML1 amphipols, three conformational states were identified, state 1 (~200 K, 26%), state 2 (~197 K, 25%) and state 3 (~167 K, 21%). Particles corresponding to state 2 and 3 were refined to 7.4 Å and 7.7 Å, respectively, with global mask applied and C4 symmetry imposed using RELION2.0. To further improve homogeneity and push resolution, further classification were performed onto particles corresponding to state 1, and the dominant group was refined to 5.8 Å finally. The map was sharpened using post-processing option of RELION with a B-factor of −400 Å^2^. For dataset of mTRPML1 nanodiscs, the particles were mainly composed by two sub-groups, particles with mTRPML1 inserting into nanodiscs (~242 K, 28%) and without nanodiscs (~376 K, 43 %). Though including less particles, the homogeneity and resolution of particle group with TRPML1 inserting into nanodiscs were obviously higher than particles without nanodiscs. The particle group with TRPML1 inserting into nanodiscs was further refined to 5.4 Å with global mask applied and C4 symmetry imposed using RELION2.0. The map was sharpened with a B-factor of −400 Å^2^. The local resolution map was calculated using ResMap (Kucukelbir et al., 2014), and displayed in Chimera (Pettersen et al., 2004).

### Model building

Model of mTRPML1 transmembrane domain was predicted on I-TASSER server (Zhang, 2008). The predicted model of transmembrane domain and crystal structure of I-II linker (5TJA) were merged, docked into the two cryo-EM maps with higher resolutions (5.8 and 5.4 Å) from mTRPML1 amphipols and mTRPML1 nanodiscs sample respectively in Chimera, and manually adjusted in Coot (Emsley et al., 2010) to acquire the atom models of state 1 from mTRPML1 amphipols and mTRPML1 nanodiscs sample. Sequence alignment and secondary structure prediction of human TRPML1 were used to aid the model building. Model refinement was performed on the main chain of the two atom models using phenix.real_space_refine. Atom model of state 1 from mTRPML1 amphipols sample was docked into cryo-EM map of state 2 and 3, and each domain of the atom model were rigid-body fitted into the two density map respectively in Chimera (Pettersen et al., 2004) to acquire the atom models of state 2 and 3.

### Quantification and statistical analysis

All reported resolutions are based on the gold-standard FSC = 0.143 criteria (Scheres and Chen, 2012), and the final FSC curve were corrected for the effect of a soft mask using high-resolution noise substitution (Chen et al., 2013). Final density maps were sharpened with a B-factor of −400 Å^2^ using RELION. Local resolution map was calculated using ResMap (Kucukelbir et al., 2014).

### Data and software availability

The coordinates of the mTRPML1 in Amphipols and Nanodiscs have been deposited in the Worldwide Protein Data Bank (http://www.rcsb.org) with the accession codes 5YDZ, 5YE1, 5YE2 and 5YE5, respectively. The corresponding maps have been deposited in the Electron Microscopy Data Bank (http://emdatabank.org) with the accession codes EMD-6823, EMD-6824, EMD-6825, and EMD-6826, respectively.

## Star Methods

### Contact for reagent and resource sharing

For reagents in this paper or more information about resource sharing, please contact the corresponding author Maojun Yang (maojunyang@tsinghua.edu.cn)

## Electronic supplementary material

Below is the link to the electronic supplementary material.

Supplementary material 1 (PDF 1496 kb)
