# Supplementary material for: Cryo-EM structures of the mammalian endo-lysosomal TRPML1 channel elucidate the combined regulation mechanism
Source: Protein Cell. 2017 Sep 21;8(11):834–47. doi: 10.1007/s13238-017-0476-5 (PMC5676595; doi:10.1007/s13238-017-0476-5)
Supplement: Supplementary file 1 — Supplementary material 1 (PDF 1496 kb) [file 13238_2017_476_MOESM1_ESM.pdf]

Supplemental Fig. and Fig. Legends

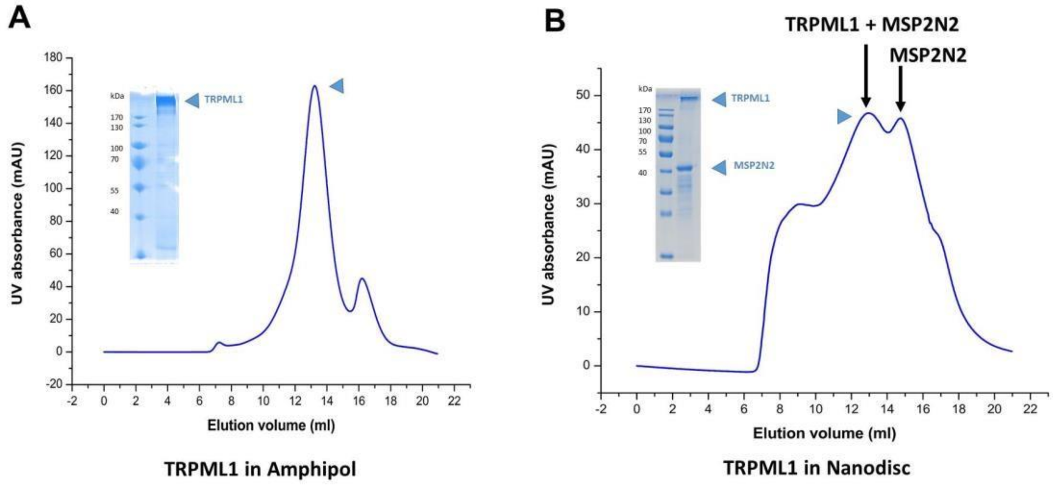

Fig. S1. Purification of mTRPML1 in Amphipols and Nanodiscs, Related to Figs. 1-5 and Methods.

A. Size-exclusion chromatography of mTRPML1 in Amphipols A8-35 on a Superose 6 column (GE Healthcare). The peaks corresponding to the TRPML1 channel are indicated with blue arrows. The mTRPML1 protein remained tetrameric even in reducing SDS-PAGE.

B. Size-exclusion chromatography of mTRPML1 in lipid nanodiscs on a Superose 6 column (GE Healthcare). Corresponding peak for TRPML1 nanodiscs was examined by SDS-PAGE indicated by blue arrow.

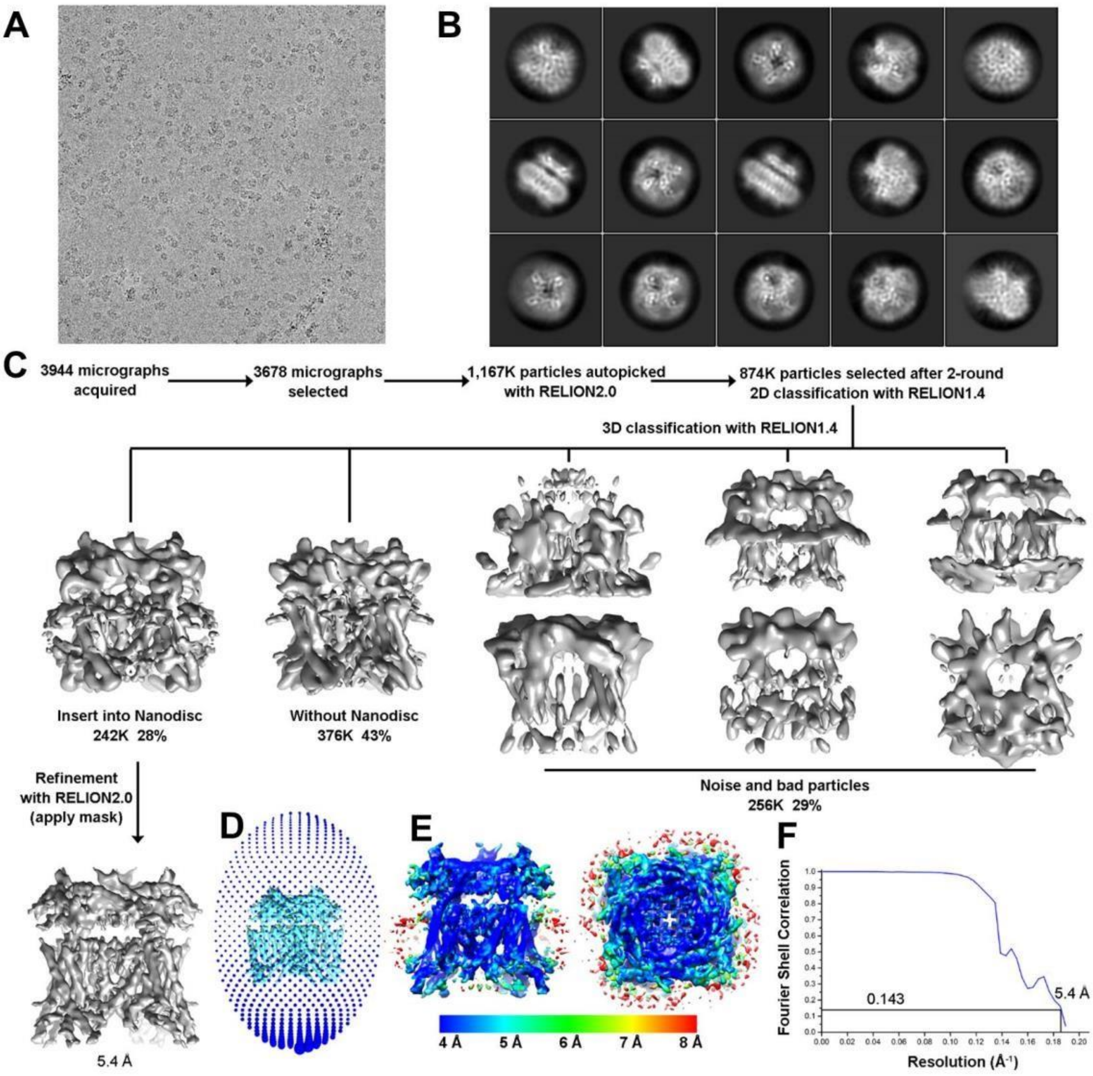

Fig. S2. mTRPML1 Structure Determination in Lipid Nanodiscs, Related to Figs. 1-3 and Methods.

A. Representative raw micrograph of mTRPML1 in nanodiscs.
B. Representative 2D class average of mTRPML1 in nanodiscs.
C. Workflow of 3D classification and subsequent refinement for the class inserted into lipid nanodiscs. For the refinement of mTRPML1 particles in nanodiscs, several available methods were tested and C4 symmetry was imposed throughout the refinement.
D. Angular distribution of the final reconstruction.
E. Local resolution map and angular distribution of the final TRPML1 nanodiscs map. Top view and side view including densities of nanodiscs are shown.
F. Gold-standard Fourier shell correlation (FSC) curves (after correction for masking effects) of the final nanodiscs map. Resolution estimation (5.4 Å) was based on the criterion of FSC 0.143 cutoff.

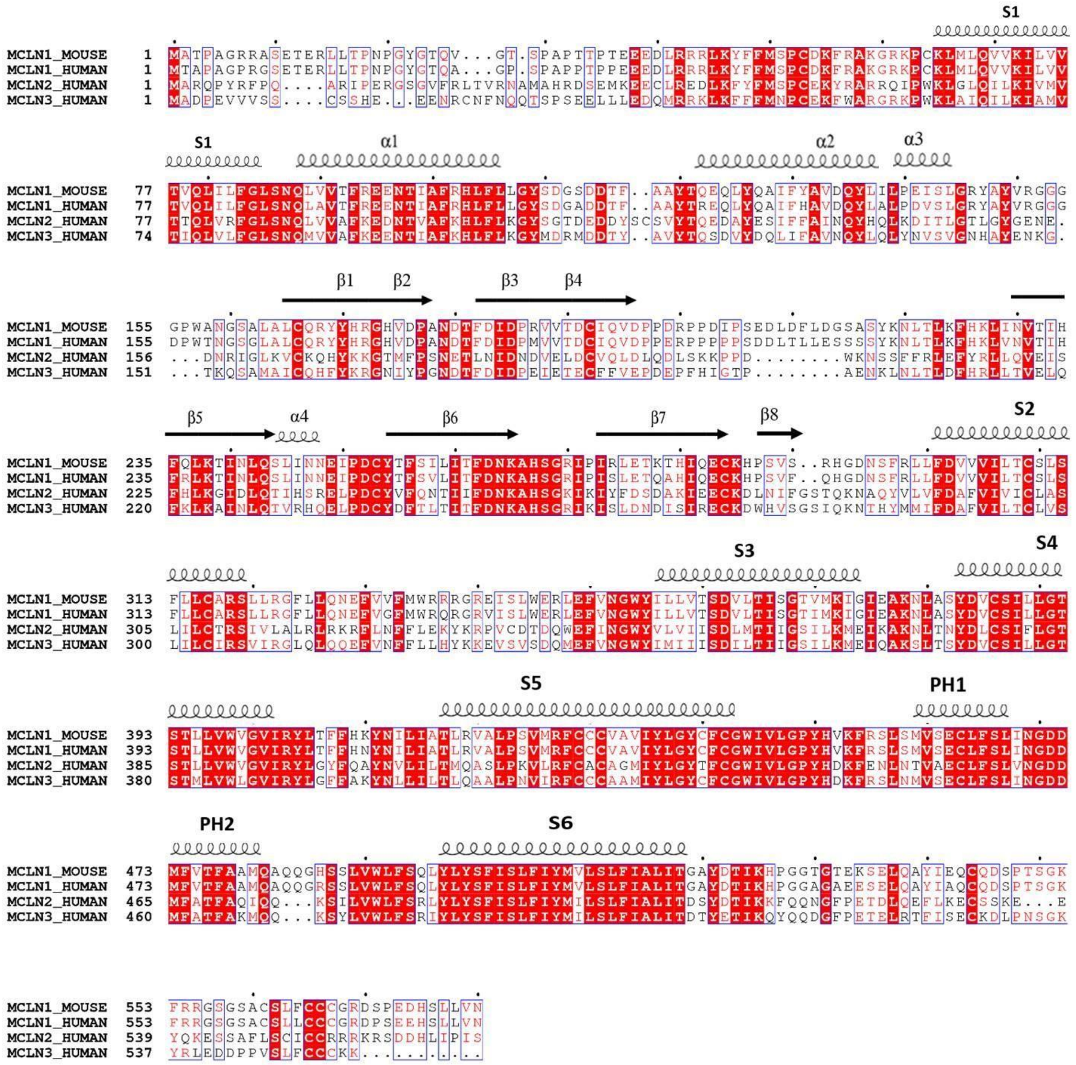

Fig. S3. Sequence Alignment of TRPML1 in Difference Species, Related to Figs. 1-4.

Sequence alignment of *Mus musculus* TRPML1, *Homo sapiens* TRPML1, *Homo sapiens* TRPML2 and *Homo sapiens* TRPML3 using ESPript 3. Secondary structure elements are indicated above the sequence, shown as arrow (β sheets), helix (α helix).

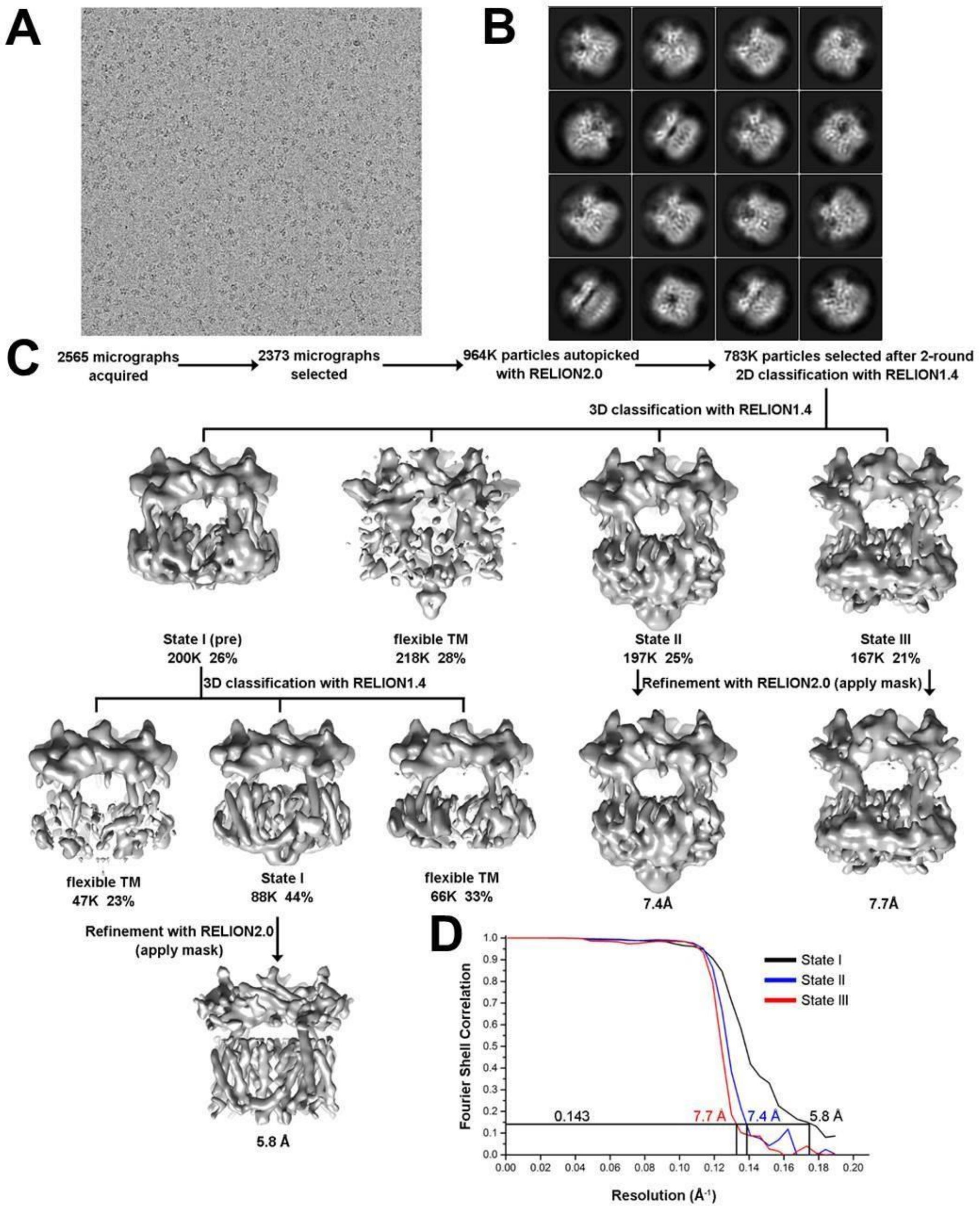

Fig. S4. Structure Determination of mTRPML1 in Amphipols A8-35, Related to Figs. 4-7 and Methods.

A. Representative raw micrograph of mTRPML1 in Amphipols.
B. Gallery of two-dimensional class averages of mTRPML1 in Amphipols.
C. Workflow of 3D classification and subsequent refinement of three individual classes. C4 symmetry was imposed throughout the refinement.
D. Gold-standard Fourier shell correlation (FSC) curves (after correction for masking effects) of mTRPML1 in three states. Resolution estimation (5.8 Å, 7.4 Å and 7.7 Å) was based on the criterion of FSC 0.143 cutoff.

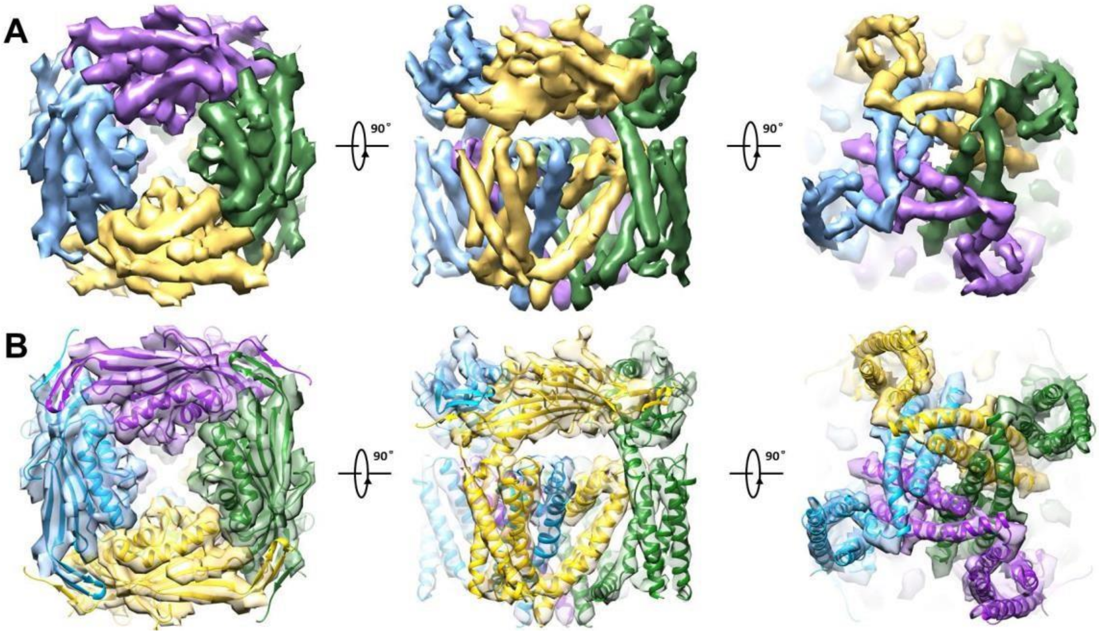

Fig. S5. Overall Structure of mTRPML1 in State 1, Related to Figs. 4-7 and Methods

A. Cryo-EM density map of mTRPML1 at a resolution of 5.8 Å in Amphipols with each subunit color-coded. Three views are shown from the endo-lysosomal lumen, side and cytosol.
B. TRPML1 model is superimposed onto the cryo-EM map with different views as panel (A) showed.

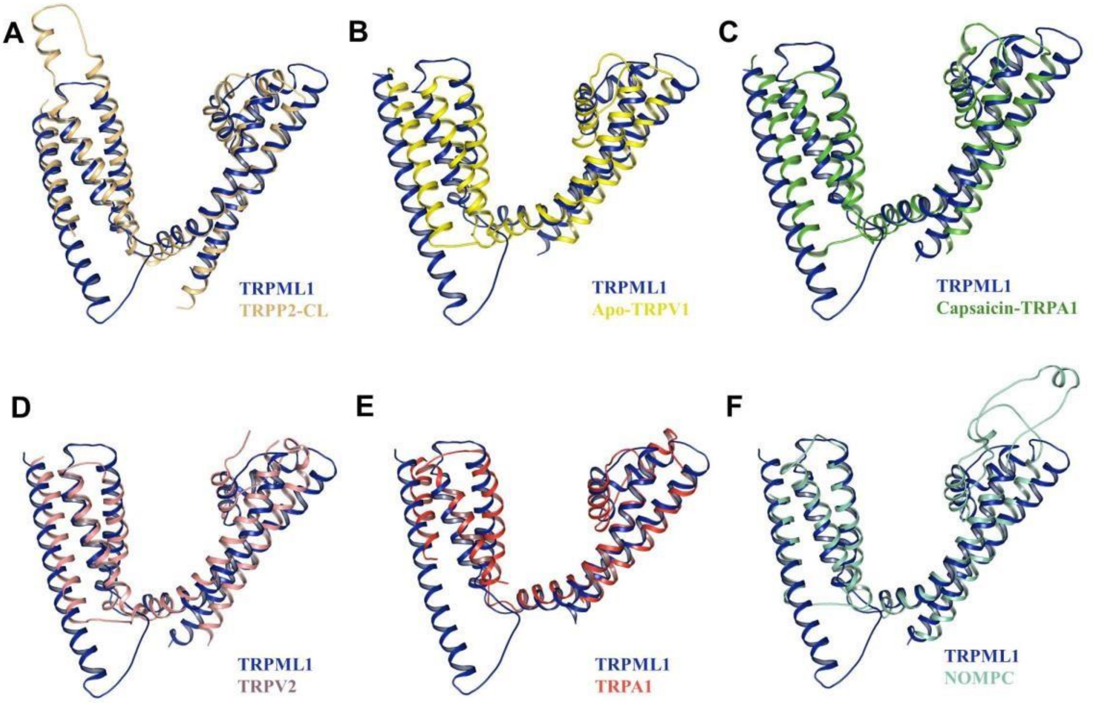

Fig. S6. Comparison of the VSD and the Filter Region in mTRPML1 with other TRP Family Channels, Related to Figs. 1 and 3.

The VSD (S2-S4 only) and pore regions (S5-P-S6) of TRPML1 (blue ribbon) was aligned with TRPP2-CL (A, PDB accession number 5T4D), Apo-TRPV1 (B, PDB accession number 3J5P), Capsaicin-TRPV1 (C, PDB accession number 5IS0), TRPV2 (D, PDB accession number 5AN8), TRPA1 (E, PDB accession number 3J9P), NOMPC (F, PDB accession number 5VKQ). TRPML1 has a long extension intervening S2 and S3, which is different from other TRP channels, while TRPP2 has a long loop between S3 and S4. Compared with TRPV1, TRPV2 and TRPA1, the distance between S2 and filter region is longer in TRPML1.

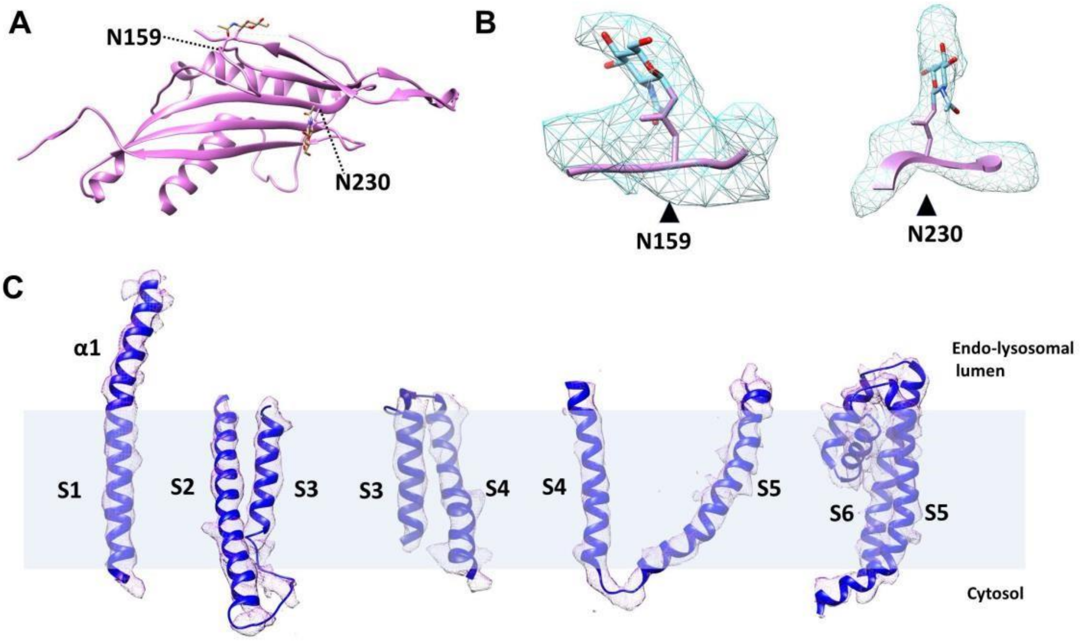

Fig. S7. N-glycan Modifications of mTRPML1 and Density Contributes to the Transmembrane Region of mTRPML1, Related to Figs. 1-6.

A. Mapping of resolved N-glycosylation sites onto the PMD structure. N159 is located at a flexible loop, while N230 is located in the antiparallel β regions.
B. N159 and N230 occurs at the consensus NX(T/S) motif where X stands for any amino acids.
C. Alanine models of transmembrane regions are displayed with their densities (mesh) superimposed. The transmembrane regions are highlighted by a light purple shade with endo-lysosomal lumen and cytosol indicated.
